# Giant Left Ventricular Aneurysmal Dilatation With Coexisting Pseudoaneurysm After Anterior STEMI: Multimodality Imaging–Guided Repair

**DOI:** 10.1016/j.jaccas.2025.105527

**Published:** 2025-10-29

**Authors:** Juan Miguel Miranda-Torrón, Manuel Carnero-Alcázar, Irene Marco, Pedro Marcos-Alberca, Luis Carlos Maroto-Castellanos

**Affiliations:** a Cardiac Surgery Department, Hospital Universitario Clínico San Carlos, Madrid, Spain; b Cardiology Department, Heart Failure Unit, Hospital Universitario Clínico San Carlos, Madrid, Spain; c Cardiology Department, Cardiovascular Imaging, Hospital Universitario Clínico San Carlos, Madrid, Spain

**Keywords:** apical aneurysm, cardiac magnetic resonance, Dor procedure, echocardiography, giant ventricular aneurysm, left ventricular aneurysm, left ventricular pseudoaneurysm multimodality imaging, myocardial infarction complications, surgical ventricular reconstruction

## Abstract

**Background:**

Left ventricular (LV) pseudoaneurysm is an uncommon but life-threatening complication after myocardial infarction.

**Case Summary:**

A 60-year-old man with untreated anterior ST-segment elevation myocardial infarction in 2021 developed a giant apical aneurysm (78 × 65 mm) and suspected pseudoaneurysm. Urgent surgery at that time revealed no rupture, and the aneurysm was left in situ. During follow-up under optimal medical therapy, multimodality imaging (transthoracic echocardiography, computed tomography, magnetic resonance imaging) documented progressive aneurysmal dilatation to 88 × 72 mm, and in 2025, a new pseudoaneurysm was identified within the aneurysmal segment. Surgical reconstruction with neo-apex formation (Fontan stitches, Dacron patch) was performed, restoring LV geometry.

**Discussion:**

This case underscores the value of serial multimodality imaging for detecting delayed pseudoaneurysm formation and guiding surgical timing. Late repair can achieve excellent outcomes when based on accurate anatomical definition and multidisciplinary decision-making.

**Take-Home Messages:**

Longitudinal multimodality imaging detects late complications and optimizes timing. Accurate anatomical definition enables complex LV reconstruction even years after myocardial infarction.

## Graphical Abstract

**Figure:**
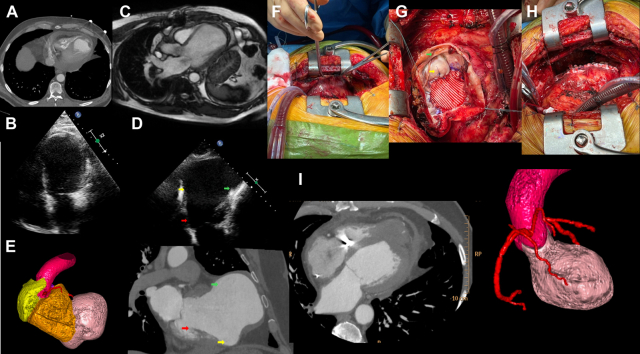

## History of Presentation

A 60-year-old man was referred for surgical evaluation of progressive left ventricular (LV) aneurysm enlargement. He reported increasing fatigue over months but denied angina or syncope. Vitals signs included blood pressure of 122/76 mm Hg, heart rate of 68 beats/min, and oxygen saturation of 97%. Physical examination showed regular rhythm, no murmurs, and no signs of heart failure. Electrocardiogram revealed persistent Q waves in the anterior leads, and laboratory tests were unremarkable.

**Take-Home Messages**

• Longitudinal multimodality imaging detects late complications and optimizes timing.
• Accurate anatomical definition enables complex LV reconstruction even years after myocardial infarction.

## Medical History

In June 2021, the patient experienced ST-segment elevation myocardial infarction that was untreated owing to late presentation. In July 2021, he was hospitalized with large left pleural effusion; imaging suggested LV aneurysm ± pseudoaneurysm. Urgent surgery found no clear rupture. Coronary angiography demonstrated a left anterior descending artery occlusion distal to the first diagonal branch. In December 2021, the patient underwent implantable cardioverter-defibrillator placement owing to an ejection fraction (EF) of 30%. He had no history of hypertension, diabetes, or smoking.

## Differential Diagnosis

The differential diagnosis included true apical LV aneurysm, LV pseudoaneurysm, pericardial effusion with tamponade, and intracavitary thrombus. Distinguishing aneurysm from pseudoaneurysm was critical for prognosis and surgical indication.

## Investigations

**Figure 1:**
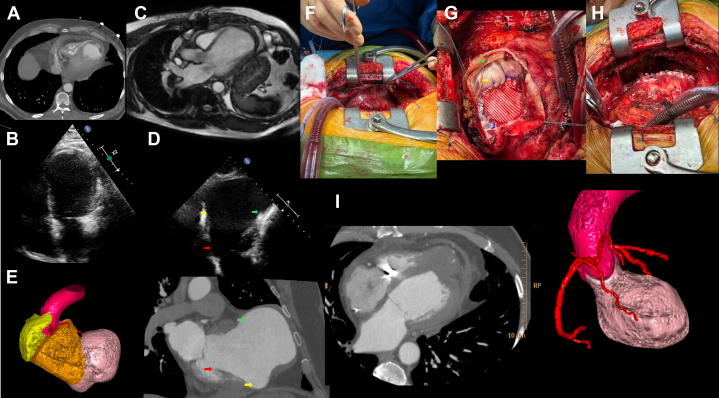
Case Presentation and Surgical Management (A) 2021 CT scan with thinning of the apical left ventricular wall with contained myocardial rupture subjective of pseudoaneurysm. (B) 2021 TTE with contrast showing aneurysmal dilatation of the left ventricle with apical thinning, absence of pericardial effusion. (C) 2021 MRI showing a dilated left ventricle with marked thinning of the anterior and anteroseptal walls, an apical aneurysm, and mural thrombus in the anterior wall. (D) 2024 TTE showing apical aneurysm and left ventricular enlargement. Shown is the transition zone between true left ventricle cavity (red arrow), aneurysm (yellow arrow) and pseudoaneurysm (green arrow). (E) 2024 CT 3-dimensional reconstruction shows the difference between normal left ventricle and pseudoaneurysm. CT scan to the right shows apical aneurysm and left ventricular enlargement. Transition zone between true left ventricle cavity (red arrow), aneurysm (yellow arrow) and pseudoaneurysm (green arrow). (F) Left ventricular aneurysm adhered to the thoracic wall. (G) Transition zone between true left ventricle cavity below Dacron patch, aneurysm (yellow arrow) and pseudoaneurysm (green arrow). (H) Left ventricle after repair. (I) Postoperative CT scan shows a nondilated left ventricle, absence of the previously seen pseudoaneurysm, presence of a Dacron patch at the neo-apex, and no pericardial effusion. CT = computed tomography; MRI = magnetic resonance imaging; TTE = transthoracic echocardiography.
• 2021 magnetic resonance imaging (MRI): LV dilation (end-diastolic volume: 245 mL, EF: 30%), transmural scar in anterior/anterior-septal walls (Figure 1C).
• 2022-2024 annual TTE and chest x-ray: gradual enlargement of aneurysm to 88 × 72 mm (Figures 1B and 1C).
• 2025 CT: the aneurysmal neck measured 68 mm, with a maximal diameter of 98 mm. A suspected pseudoaneurysmal cavity was identified at the inferior margin of the aneurysm, measuring 40 × 20 mm. The indexed LV end-diastolic volume was 482 mL including the aneurysmal component and 285 mL when excluded, confirming the extent of geometric distortion (Figure 1E).
• 2025 MRI: EF of 28%, large scar burden, confirmed pseudoaneurysm.

Findings on serial multimodality imaging were as follows:

• 2021 TTE: apical thinning, aneurysmal dilation, moderate pericardial effusion.
• 2021 CT: contained rupture suggestive of pseudoaneurysm (78 × 65 mm) (Figure 1A).

## Management (Medical/Interventions)

Medical management included beta-blocker, angiotensin-converting enzyme inhibitor, mineralocorticoid antagonist, loop diuretic, and antiplatelet therapy. No ischemia-driven revascularization was performed owing to distal left anterior descending artery occlusion and nonviable myocardium.

Regarding surgical management, in April 2025 the patient underwent median sternotomy, and the aneurysm and pseudoaneurysm were identified. A neo-apex was created with Fontan stitches at the transition to a viable myocardium; Dacron patch endoventriculoplasty (Dor technique) excluded diseased segments (Figures 1F, 1G, and 1H). The postoperative course was uneventful; the patient was extubated in the operating room, and he remained in the intensive care unit for 48 hours.

## Outcome and Follow-Up

The patient was discharged on postoperative day 7. Postoperative CT and TTE confirmed complete exclusion of aneurysmal/pseudoaneurysmal cavities and restored LV geometry, and quantitative analysis revealed a significantly reduced LV end-systolic volume of 104 mL, compared with 482 mL preoperatively (Figure 1I). At the 1-month follow-up, he was assessed as NYHA functional class I, with no arrhythmias and stable EF. Imaging was scheduled at 6 and 12 months.

## Discussion

Mechanical complications after myocardial infarction, though rare today, may present late in patients who remain untreated during the acute phase.^1^ This case illustrates the progression of a large apical aneurysm into a pseudoaneurysm, likely due to a contained rupture undetected during initial surgery. Dense adhesions and lack of active bleeding often obscure true pathology.

Distinguishing between aneurysm and pseudoaneurysm is critical, as the latter carries a high risk of rupture and mandates surgical repair. In contrast, true aneurysms can often be managed conservatively.^2^

Serial multimodality imaging—including TTE, CT, and MRI—was essential for timely re-evaluation.^3^ Despite guideline-directed therapy, progressive remodeling and symptom worsening prompted successful delayed surgical repair. Careful follow-up and multidisciplinary evaluation enabled a favorable outcome.

This case is unique in that it documented the delayed development of an LV pseudoaneurysm within a pre-existing aneurysmal cavity, despite stable imaging and medical therapy over 4 years. The integration of serial multimodality imaging, an initially conservative surgical decision, and a successful late reconstruction provides valuable clinical and educational insight into the long-term management of mechanical complications after myocardial infarction.

## Conclusions

Chronic LV aneurysms can evolve into pseudoaneurysms years after myocardial infarction. Serial multimodality imaging is indispensable for timely detection and safe surgical repair.

## Funding Support and Author Disclosures

The authors have reported that they have no relationships relevant to the contents of this paper to disclose.
